# One-Day Versus Three-Day Dexamethasone with NK1RA for Patients Receiving Carboplatin and Moderate Emetogenic Chemotherapy: A Network Meta-analysis

**DOI:** 10.1093/oncolo/oyac060

**Published:** 2022-04-15

**Authors:** Daichi Watanabe, Hirotoshi Iihara, Hironori Fujii, Akitaka Makiyama, Shohei Nishida, Akio Suzuki

**Affiliations:** Department of Pharmacy, Gifu University Hospital, Gifu, Japan; Department of Pharmacy, Gifu University Hospital, Gifu, Japan; Department of Pharmacy, Gifu University Hospital, Gifu, Japan; Cancer Center, Gifu University Hospital, Gifu, Japan; Department of Pharmacy, Gifu University Hospital, Gifu, Japan; Department of Pharmacy, Gifu University Hospital, Gifu, Japan

**Keywords:** antiemetics, neurokinin, 1 receptor antagonists, dexamethasone, nausea, vomiting, antineoplastic agents, carboplatin, network meta, analysis

## Abstract

**Background:**

The dexamethasone (DEX)-sparing strategy, which limits administration of DEX to day one, is reportedly non-inferior to conventional antiemetic regimens comprising multiple-day DEX. However, the usefulness of the DEX-sparing strategy in triplet antiemetic prophylaxis (neurokinin-1 receptor antagonist [NK1RA] + serotonin receptor antagonist [5HT3RA] + DEX) for carboplatin and moderate emetogenic chemotherapy (MEC) has not been clarified.

**Patients and Methods:**

We systematically reviewed randomized controlled trials that examined the efficacy of antiemetics for preventing chemotherapy-induced nausea and vomiting associated with carboplatin and MEC. We conducted a network meta-analysis to compare the antiemesis efficacy of three-day DEX with NK1RA (3-DEX + NK1RA) and one-day DEX with NK1RA (1-DEX + NK1RA). The primary outcome was complete response during the delayed phase (CR-DP). The secondary outcome was no nausea during the delayed phase (NN-DP).

**Results:**

Seventeen trials involving 4534 patients were included. The proportion who experienced CR-DP was 82.5% (95% credible interval [CI], 73.9-88.6) and 73.5% (95% CI, 62.8-80.9) among those who received 3-DEX + NK1RA and 1-DEX + NK1RA, respectively. There was no significant difference between the two regimens. However, 3-DEX + NK1RA tended to be superior to 1-DEX + NK1RA, with an absolute risk difference of 9.0% (95% CI, −2.3 to 21.1) in CR-DP and 24.7% (95% CI: −14.9 to 54.6) in NN-DP. 3-DEX + NK1RA also tended to be superior to 1-DEX + NK1RA in patients who received carboplatin-based chemotherapy, for whom the absolute risk difference was 12.3% (95% CI, −3.2 to 30.7).

**Conclusions:**

Care is needed when administering the DEX-sparing strategy in combination with NK1RA to patients receiving carboplatin and non-carboplatin MEC.

## Teaser

Minimizing chemotherapy-induced nausea and vomiting (CINV) is important to improve patients’ quality of life and ensure they continue chemotherapy. This meta-analysis compared one-day and three-day dexamethasone in combination with NK1RA for preventing CINV associated with carboplatin and non-carboplatin moderate emetogenic chemotherapy.

**Implications for Practice**

This study demonstrated that a three-day dose of dexamethasone (DEX) with neurokinin-1 receptor antagonist (NK1RA) was nonsignificantly superior to a one-day dose of DEX with NK1RA as prophylaxis for delayed-onset nausea and vomiting. The absolute risk difference in complete response during the delayed phase was 9.0% among patients who received moderate emetogenic chemotherapy (MEC), and 12.3% among patients who received carboplatin. Therefore, we propose that the majority of patients, with the exception of patients who should receive minimal DEX, such as those intolerant to corticosteroids, should receive DEX beyond day one of treatment when receiving non-carboplatin MEC or carboplatin.

## Introduction

Chemotherapy-induced nausea and vomiting (CINV) often reduces quality of life, treatment adherence, treatment efficacy, and curability in patients receiving chemotherapy. It is important to minimize the incidence of CINV to improve patients’ quality of life and ensure they continue chemotherapy.

Although dexamethasone (DEX) is effective for suppressing CINV, short-term steroid use as antiemetic prophylaxis for chemotherapy can cause steroid-induced adverse events such as elevated blood glucose levels,^1^ reduced bone mineral density,^2^ and other symptoms.^3^ Several studies and meta-analyses have shown that, as an antiemetic prophylaxis, the DEX-sparing strategy, which involves limiting administration of DEX to day one, in combination with palonosetron is non-inferior to multiple-day DEX.^4-10^ In terms of high emetogenic chemotherapy (HEC), a randomized phase III trial showed a one-day dose of DEX is non-inferior to a three-day dose of DEX in combination with neurokinin-1 receptor antagonist (NK1RA) and palonosetron in patients receiving anthracycline plus cyclophosphamide therapy. On the other hand, a one-day dose of DEX was indicated to be insufficient to control CINV in patients receiving cisplatin-containing regimen.^11^ Thus, global guidelines recommend continuous administration of DEX in the delayed phase (DP) for patients receiving cisplatin-containing chemotherapy.^12-15^

Recent guidelines classify carboplatin from a moderate emetogenic chemotherapy (MEC) to HEC,^12-15^ and recommend that patients receiving carboplatin area under the curve ≥ 4 mg/mL/min be offered triplet antiemetic prophylaxis, which combines a NK1RA with a 5-hydroxytryptamine-3 receptor antagonist (5HT3RA) and DEX.^12-15^ For patients receiving carboplatin, American Society of Clinical Oncology and Multinational Association of Supportive Care in Cancer (MASCC)/European Society for Medical Oncology (ESMO) guidelines recommend combining a one-day DEX dose with NK1RA and 5HT3RA.^14,15^ However, the recently updated National Comprehensive Cancer Network (NCCN) guideline recommends the DEX-sparing strategy be limited to patients with few specific risk factors for CINV or intolerance to corticosteroids.^12^ A recent trial by Iihara et al. showed that CINV associated with carboplatin occurs in the DP rather than the acute phase.^16^ A propensity score matching retrospective cohort study (*N* = 56) using data from a prospective observational study in clinical practice showed that a three-day dose of DEX was significantly superior to a one-day dose of DEX in triplet antiemetic prophylaxis for preventing nausea in patients receiving carboplatin.^17^

However, no randomized controlled trial (RCT) to date has examined the usefulness of combining DEX-sparing strategy with NK1RA in patients receiving carboplatin. For patients receiving non-carboplatin MEC, global guidelines recommend adding NK1RA to DEX and 5HT3RA as one antiemetic prophylaxis option,^12,13^ while the usefulness of combining the DEX-sparing strategy with NK1RA has not been established for these patients either. It is necessary to clarify the role of DEX in preventing delayed-onset CINV in triple antiemetic prophylaxis for patients receiving carboplatin and non-carboplatin MEC.

Therefore, we conducted a network meta-analysis (NMA) to compare one-day and three-day DEX in combination with NK1RA for preventing CINV associated with carboplatin and non-carboplatin MEC.

## Patients and Methods

### Objectives

The present study aimed to compare one-day versus three-day DEX in combination with NK1RA and 5HT3RA for preventing CINV associated with carboplatin and non-carboplatin MEC described in the most recent NCCN guideline.^12^ The present NMA was prospectively registered (PROSPERO registration number: CRD42021256346) and was designed according to the Preferred Reporting Items for Systematic Reviews and Meta-Analyses guidelines.

### Inclusion and Exclusion Criteria

Randomized (phase II or III) clinical trials were included if they were published in English. Trials of interest were those that compared the efficacy of globally available antiemetics in adult patients with cancer receiving carboplatin or non-carboplatin MEC. Trials that compared two of the following antiemetic strategies were included: (1) three-day DEX with NK1RA (3-DEX + NK1RA), (2) one-day DEX with NK1RA (1-DEX + NK1RA), (3) three-day DEX (3-DEX), and (4) one-day DEX (1-DEX). Studies that used drugs other than DEX, 5HT3RA, and NK1RA for antiemetic prophylaxis, such as olanzapine or metoclopramide, were excluded.

### Outcome Measures

The primary outcome was complete response (CR; no emesis and no rescue medication) during the DP (24-120 h after chemotherapy initiation). The secondary outcome was no nausea during the DP (NN-DP). For the primary outcome, subgroup analysis was performed in patients who received carboplatin-based chemotherapy and those who received a three-day dose of first-generation 5HT3RA or single dose of palonosetron (long 5HT3RA). All outcome variables were extracted in the first planned chemotherapy cycle.

### Study Selection and Data Extraction

We systematically searched for eligible RCTs published through May 15, 2021 using PubMed and Ovid-MEDLINE. We used a combination of the terms “chemotherapy-induced nausea and vomiting,” “moderately emetogenic chemotherapy,” “steroid-sparing,” “neurokinin-1 receptor antagonists,” “fosaprepitant,” “ezlopitant,” “netupitant,” “rolapitant,” and “aprepitant” to find relevant articles (see Supplementary Table S1 for details). An additional search through the reference lists of relevant reviews and meta-analyses was also conducted. Two reviewers (D.W. and H.I.) independently assessed the abstracts of all relevant studies to confirm their eligibility and extracted details from the included studies, including the study design, study population characteristics, inclusion criteria, outcome measures, chemotherapy regimen, and details of the antiemetic regimen. Decisions made by each of the two reviewers were compared, and any disagreement was resolved through consensus between the two reviewers.

### Risk of Bias Assessment

Two reviewers (D.W. and H.I.) independently assessed the risk of bias due to the randomization process, deviations from the intended interventions, missing outcome data, measurement of the outcome, selection of the reported results, and other biases of the included studies (Supplementary Table S2) using the Revised Cochrane risk-of-bias tool for randomized trials (RoB 2) by The Cochrane Collaboration (http://www.cochrane.de). Any disagreement was resolved through consensus between the two reviewers.

### Statistical Analysis

An arm-based NMA using Bayesian methods was conducted to compare multiple antiemetic strategies. NMA enables the direct comparison of treatments in individual trials and indirect comparison between trials simultaneously.^18^ In particular, an arm-based approach can be used estimate the population-averaged treatment-specific event rate. The proportions of CR-DP and NN-DP in each antiemetic strategy were aggregated using the nma.ab.bin function in the R package, pcnetmeta.^19^ The statistical heterogeneity in each treatment arm was evaluated using the I2 statistic with the metaprop function in the R package, meta. An I2 > 50% indicated statistically significant heterogeneity (Supplementary Table S3). A random effects model was used to consider heterogeneity, and the correlations between treatments were assumed to be different. Final estimation routines used 3 chains of 10 000 burn-in iterations, with 50 000 estimation iterations without thinning, resulting in 120 000 iterations for analysis. The results of NMA were estimated using posterior median with corresponding 95% credible intervals (CIs), which can be interpreted in the same manner as 95% confidence intervals. Statistical significance was indicated when the lower limit of one 95% CI exceeded the upper limit of the other 95% CI.

## Results

### Eligible Studies and Characteristics

**Figure 1.**
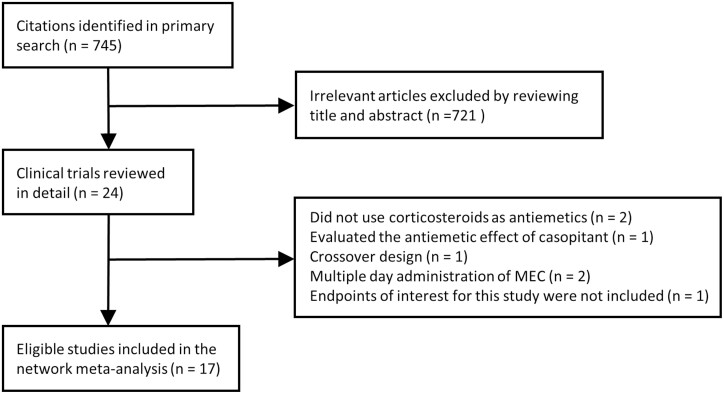
PRISMA diagram. PRISMA, Preferred Reporting Items for Systematic Reviews and Meta-Analyses.

Figure 1 shows a flow diagram of the study selection process. Among the 745 published papers on CINV identified by searching databases, we focused on 24 potentially relevant randomized control trials. We further excluded several trials because they did not use corticosteroids as an antiemetic prophylaxis (*n* = 2)^20,21^; used casopitant, which is not approved by the U.S. Food and Drug Administration, as an antiemetic prophylaxis (*n* = 1)^22^; used a crossover design and did not report the outcomes of the first chemotherapy  (*n* = 1)^23^; examined chemotherapy including multiple day administration of MEC (*n* = 2)^24,25^; or did not examine the endpoints of interest for this study (*n* = 1).^26^ Ultimately, 17 trials were included in our analyses.^5,27-42^ One article^43^ was a post-hoc analysis of another eligible study^38^; the former was used for subgroup analyses.

Table 1 summarizes the characteristics of all included trials. Eight trials compared 3-DEX + NK1RA and 3-DEX,^27,30,31,33,34,36,39,40^ five trials compared 1-DEX + NK1RA and 1-DEX,^32,37,38,41,42^ and four trials compared 3-DEX and 1-DEX,^5,28,29,35^ while no trials directly compared 3-DEX + NK1RA with 1-DEX + NK1RA (Supplementary Figure S1). Of the 4534 patients included this NMA, 11.0% (*n* = 499) received 3-DEX + NK1RA, 31.7% (*n* = 1438) received 1-DEX + NK1RA, 18.5% (*n* = 839) received 3-DEX, and 38.8%  (*n* = 1758) received 1-DEX. The proportion of patients who received carboplatin-based chemotherapy was 49.6%  (*n* = 2250). Among those administered NK1RAs, 57.5%  (*n* = 1113) received aprepitant, 25.9% (*n* = 502) received fos-aprepitant, and 16.6% (n = 322) received rolapitant. Among those administered 5HT3RAs, 42.4% (*n* = 1923) received a first-generation 5HT3RA on day one only, 35.4% (*n* = 1603) received a first-generation 5HT3RA from day one to three, and 19.7% (*n* = 895) received palonosetron. The type of 5HT3RA administered to the remaining 2.5% (*n* = 113) was unclear.

**Table 1.** Study characteristics and patient demographics of studies included in the network meta-analysis

Study name	Major chemotherapy regimen	NK1RA	5HT3RA	DEX	*N* (male/female)	Carboplatin-based regimen (%)	CR-DP	NN-DP
Aridome_2016	Oxaliplatin-based	APR	Any 5HT3RA	3-day	59 (34/25)	0	47/59	38/59
		—	Any 5HT3RA	3-day	54 (30/24)	0	43/54	37/54
Celio_2011	Carboplatin-based Oxaliplatin-based Irinotecan-based	—	Palo	3-day	100	16 (16.0)	76/100	NA
		—	Palo	1-day	111	21 (18.9)	79/111	NA
Furukawa_2015	Carboplatin-based	—	Palo	3-day	39 (0/39)	39 (100)	30/39	25/39
		—	Palo	1-day	43 (0/43)	43 (100)	30/43	26/43
Ito_2014	Carboplatin-based	APR	First-generation 5HT3RA day 1	3-day	67 (56/11)	67 (100)	54/66	35/66
		—	First-generation 5HT3RA day 1	3-day	67 (54/13)	67 (100)	46/67	29/67
Kaushal_2015	Carboplatin-based	APR	Palo	3-day	30 (29/1)	30 (100)	25/30	23/30
		—	OND day 1-3	3-day	30 (23/7)	30 (100)	16/30	13/30
Kim_2017	Carboplatin-based Oxaliplatin-based Irinotecan-based	APR	OND day 1	1-day	237 (129/108)	156 (65.8)	176/237	NA
		—	OND day 1-3	1-day	243 (134/109)	156 (64.2)	173/243	NA
Komatsu_2015	Oxaliplatin-based Irinotecan-based	—	Palo	3-day	154 (87/67)	19 (12.3)	100/154	NA
		—	Palo	1-day	151 (86/65)	18 (11.9)	101/151	NA
Kusagaya_2015	Carboplatin-based	APR	Palo	3-day	41 (29/12)	41 (100)	33/41	NA
		—	Palo	3-day	39 (28/11)	39 (100)	30/39	NA
Maehara_2015	Carboplatin-based	APR	GRA day 1	3-day	11 (0/11)	11 (100)	11/11	10/11
		—	GRN day 1	3-day	12 (0/12)	12 (100)	8/12	2/12
Matsuura_2015	Carboplatin-based	—	Palo	3-day	53 (0/53)	53 (100)	36/53	NA
		—	Palo	1-day	56 (0/56)	56 (100)	34/56	NA
Nishimura_2015	Oxaliplatin-based	APR	5HT3RA day 1	3-day	207 (126/81)	0	159/187	124/187
		—	5HT3RA day 1	3-day	206 (126/80)	0	138/183	113/183
Rapoport_2010	Carboplatin-based Irinotecan-based Oxaliplatin-based Other non-AC MEC	APR	OND day 1	1-day	226	NA^a^	172/226	NA
		—	OND day 1-3	1-day	203	NA^a^	140/203	NA
Schwartzberg_2015	Carboplatin-based Irinotecan-based Oxaliplatin-based Other non-AC MEC	ROL	GRN day 1-3	1-day	322	191 (59.3)	245/322	NA
		—	GRN day 1-3	1-day	307	209 (68.1)	196/307	NA
Sugimori_2017	Carboplatin-based	APR	Palo	3-day	39 (0/39)	39 (100)	38/39	27/39
		—	Palo	3-day	39 (0/39)	39 (100)	32/39	25/39
Tanioka_2013	Carboplatin-based	APR	GRN day 1	3-day	45 (0/45)	44 (97.8)	28/45	NA
		—	GRN day 1	3-day	46 (0/46)	45 (97.8)	24/46	NA
Weinstein_2016	Carboplatin-based Oxaliplatin-based	FAPR	OND day 1	1-day	502 (204/298)	257 (51.2)	396/502	NA
		—	OND day 1-3	1-day	498 (205/293)	256 (51.4)	341/498	NA
Yahata_2016	Carboplatin-based	APR	First-generation 5HT3RA day 1	1-day	155 (0/155)	155 (100)	96/151	61/151
		—	First-generation 5HT3RA day 1	1-day	152 (0/152)	152 (100)	72/146	49/146

Data could not be extracted from among the other chemotherapy regimens.

Abbreviations: 5HT3RA, serotonin receptor antagonist; APR, aprepitant; CR-DP, complete response during delayed phase; DEX, dexamethasone; FAPR, fos-aprepitant; GRN, granisetron; NA, not available; NK1RA, neurokinin-1 receptor antagonist; NN-DP, no nausea during delayed phase; OND, ondansetron; Palo, palonosetron; ROL, rolapitant.

### Proportion Experiencing CR-DP and NN-DP in Each Antiemetic Regimen

**Figure 2.**
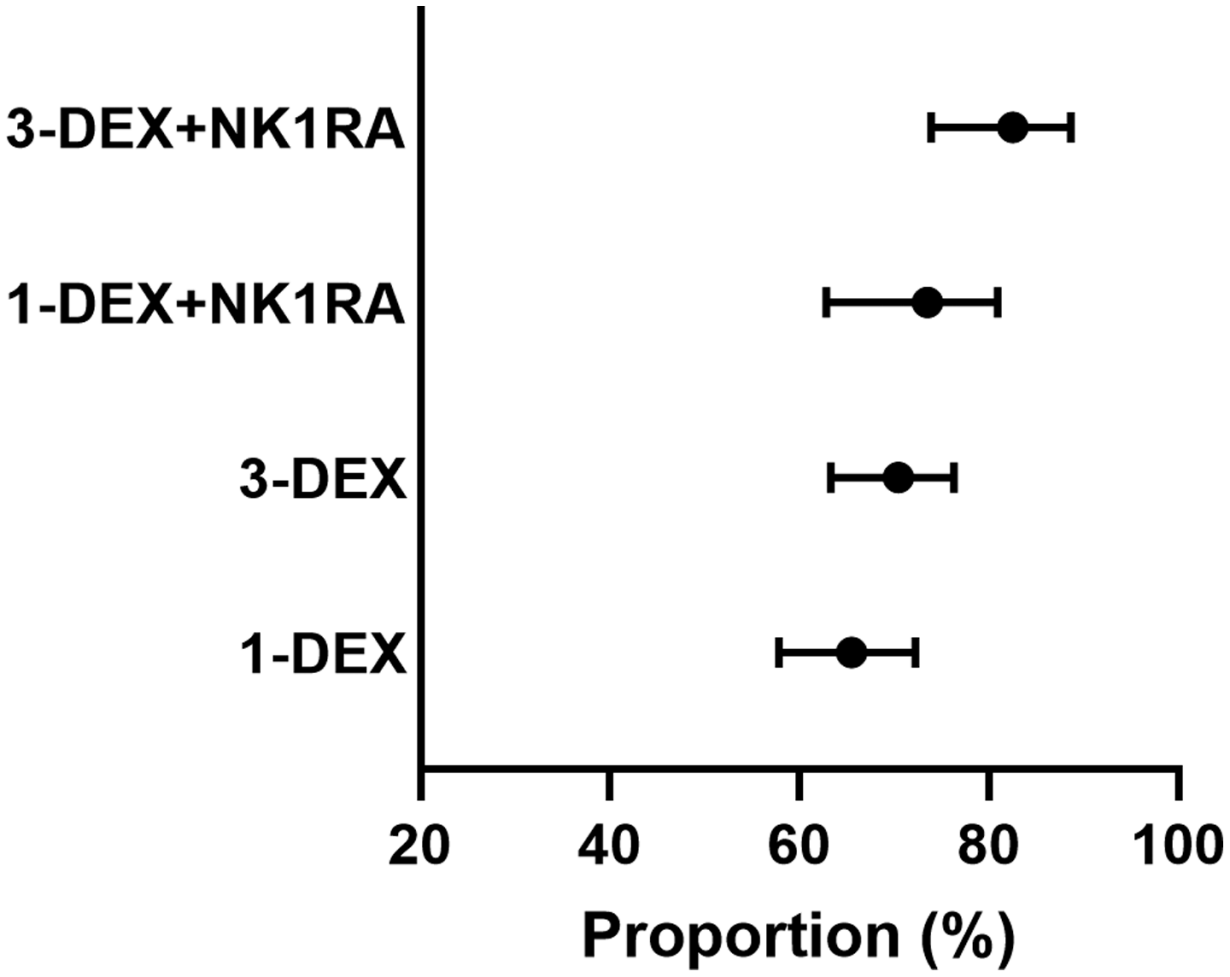
Proportion of patients who experienced complete response during the delayed phase in each antiemetic regimen among the entire population.

**Figure 3.**
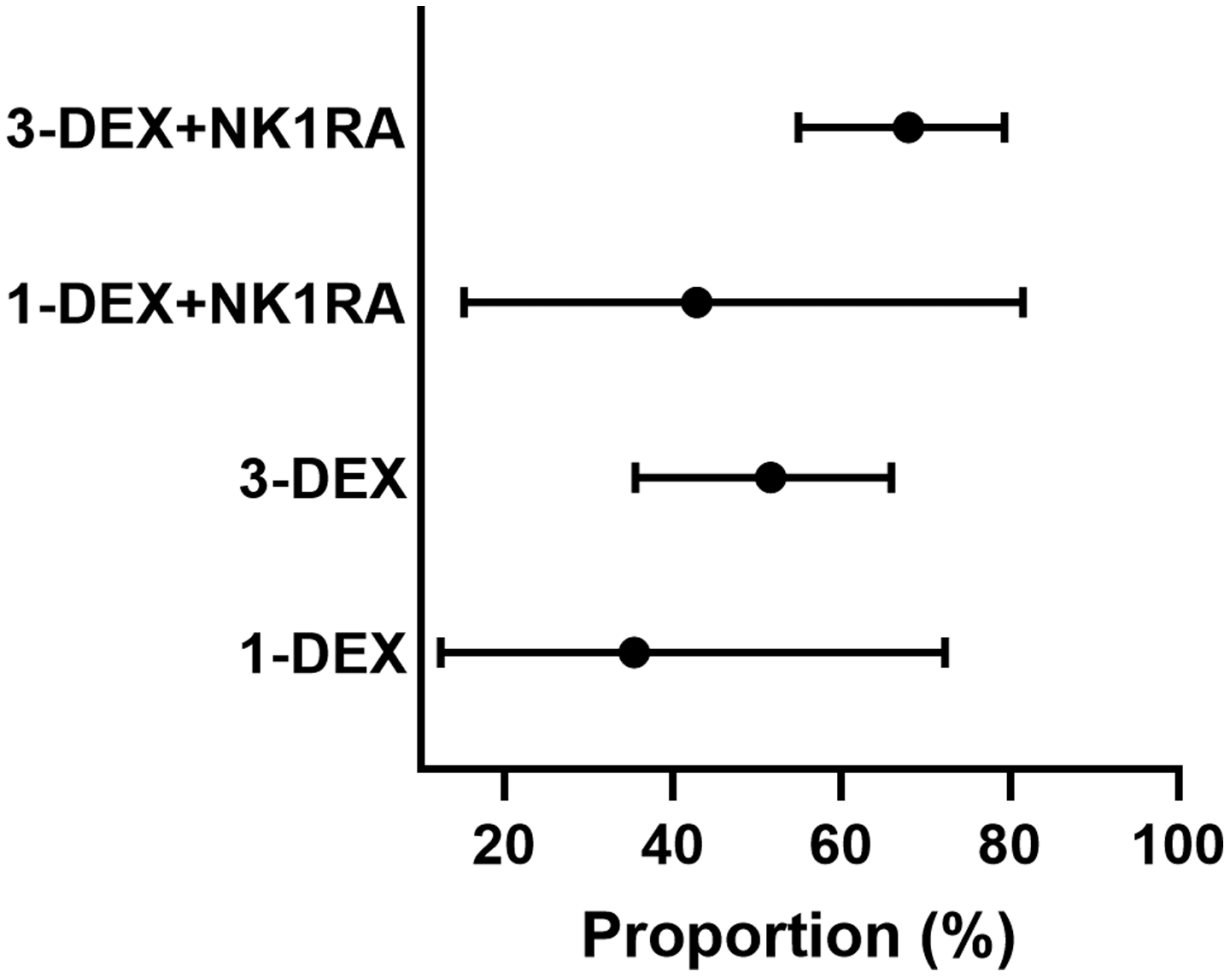
Proportion of patients who experienced no nausea during the delayed phase in each antiemetic regimen among the entire population.

The proportion of patients who experienced CR-DP was 82.5% (95% CI, 73.9-88.6), 73.5% (95% CI, 62.8-80.9), 70.4% (95% CI: 63.3-76.3), and 65.5% (95% CI, 57.8-72.2) among those who received 3-DEX + NK1RA, 1-DEX + NK1RA, 3-DEX, and 1-DEX, respectively (Figure 2). Meanwhile, the proportion who experienced NN-DP was 67.9% (95% CI, 54.9-79.3), 42.8% (95% CI, 15.2-81.5), 51.6% (95% CI, 35.5-65.9%), and 35.4% (95% CI, 12.4-72.3) among patients who received 3-DEX + NK1RA, 1-DEX + NK1RA, 3-DEX, and 1-DEX, respectively (Figure 3).

**Figure 4.**
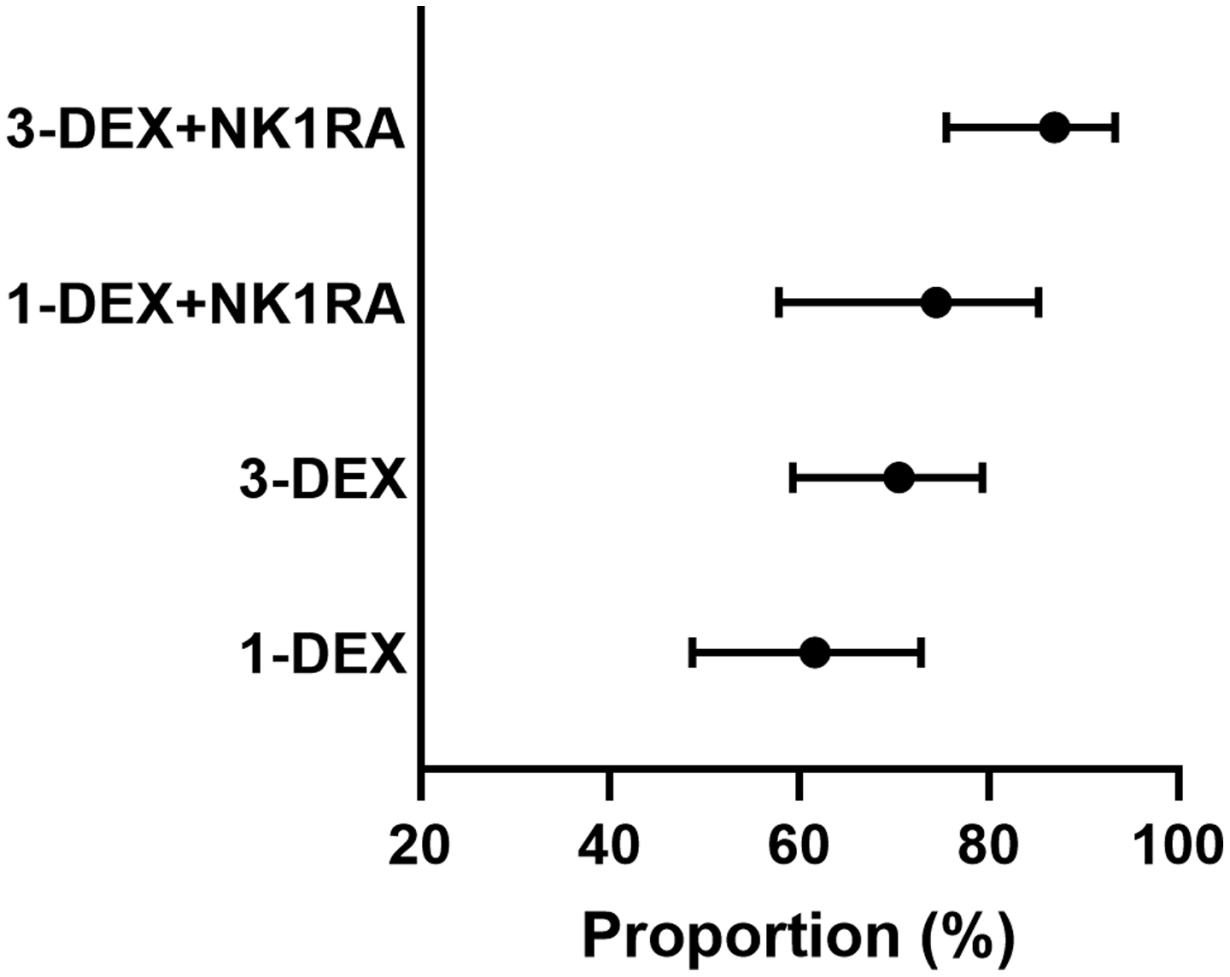
Proportion of patients who achieved complete response during the delayed phase in each antiemetic regimen among those who received a carboplatin-based regimen.

**Figure 5.**
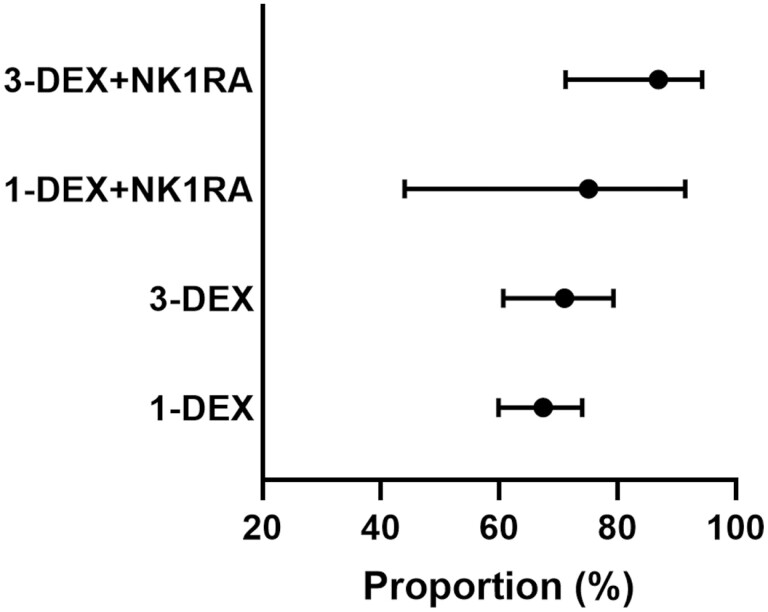
Proportion of patients who achieved complete response during the delayed phase in each antiemetic regimen among those treated with a three-day dose of first-generation 5HT3RA or single dose of palonosetron. 5HT3RA, 5-hydroxytryptamine-3 receptor antagonist.

Among patients who received carboplatin-based chemotherapy, the proportion who experienced CR-DP was 86.9% (95% CI, 75.5-93.3), 74.4% (95% CI, 57.8-85.2), 70.5% (95% CI, 59.3-79.3), and 61.6% (95% CI, 48.7-72.8) of those who received 3-DEX + NK1RA, 1-DEX + NK1RA, 3-DEX, and 1-DEX, respectively (Figure 4). Meanwhile, among patients who received long 5HT3RA, the proportion who experienced CR-DP was 86.9% (95% CI, 71.2-94.3), 75.1% (95% CI, 44.0-91.4), 71.0% (95% CI, 60.7-79.3), and 67.4% (95% CI, 59.9-74.0) of those who received 3-DEX + NK1RA, 1-DEX + NK1RA, 3-DEX, and 1-DEX, respectively (Figure 5).

### Pairwise Comparison of 3-DEX + NK1RA and 1-DEX + NK1RA

**Figure 6.**
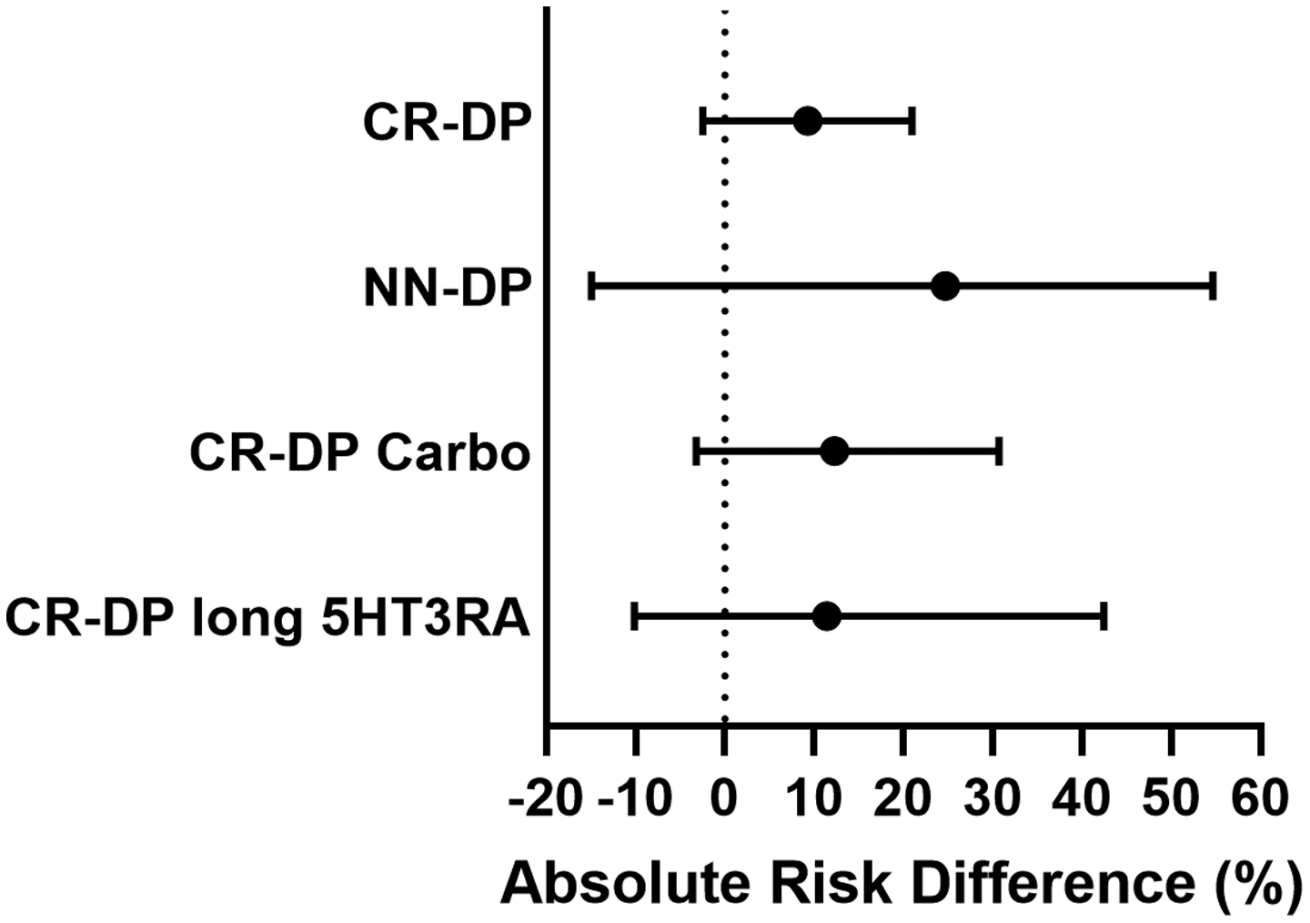
Pairwise comparison of 3-DEX + NK1RA and 1-DEX + NK1RA. Absolute risk difference greater than 1 suggests that 3-DEX + NK1RA is more efficient than 1-DEX + NK1RA. 1-DEX + NK1RA, one-day dexamethasone with neurokinin-1 receptor antagonist; 3-DEX + NK1RA, three-day dexamethasone with neurokinin-1 receptor antagonist.

Figure 6 shows the results of a pairwise comparison of 3-DEX + NK1RA and 1-DEX + NK1RA in CR-DP, NN-DP, and CR-DP in patients who received carboplatin-based chemotherapy and in CR-DP in patients who received long 5HT3RA. While there were no significant differences in any outcome between 3-DEX + NK1RA and 1-DEX + NK1RA, 3-DEX + NK1RA tended to be superior to 1-DEX + NK1RA. The absolute risk difference between 3-DEX + NK1RA and 1-DEX + NK1RA was 9.0% (95% CI, −2.3 to 21.1), 24.7% (95% CI, −14.9 to 54.6), 12.3% (95% CI, −3.2 to 30.7), and 11.4% (95% CI, −10.1 to 42.4) in CR-DP, NN-DP, and CR-DP in patients who received carboplatin-based chemotherapy and in CR-DP in patients who received long 5HT3RA, respectively.

## Discussion

We performed a systematic review and NMA to indirectly compare the antiemetic effects of 3-DEX + NK1RA and 1-DEX + NK1RA in patients receiving carboplatin and non-carboplatin MEC. Although we found no statistically significant differences, 3-DEX + NK1RA showed a tendency toward being superior to 1-DEX + NK1RA, and the absolute risk difference between 3-DEX + NK1RA and 1-DEX + NK1RA was 9.0% and 24.7% in CR-DP and NN-DP, respectively. These results suggest that continuous administration of DEX beyond day one may increase the benefits of prophylaxis for delayed-onset CINV in patients receiving carboplatin and non-carboplatin MEC.

Among patients who received carboplatin, we found that 3-DEX + NK1RA was nonsignificantly superior to 1-DEX + NK1RA in CR-DP, and that the absolute risk difference was 12.3%, which is clinically meaningful to the patient according to MASCC/ESMO.^44^ Tamura et al. reported that the CINV incidence and severity associated with cisplatin and MEC comprising mainly carboplatin and oxaliplatin peaked on days four to five.^45^ Iihara et al. reported that administration of carboplatin led to a high incidence of CINV that continued for more than seven days, with the severity peaking on days four to five with or without aprepitant.^16^ A propensity score matching retrospective cohort study that compared one-day versus three-day DEX with aprepitant and 5HT3RA for the prevention of CINV associated with carboplatin found that three-day DEX was significantly superior to one-day DEX for preventing nausea (82.5% vs. 44.4%). The study additionally found that the average severity of nausea was beyond “moderate” from day two in the one-day DEX group compared to day five in the three-day DEX group.^17^ Thus, CINV associated with carboplatin during the delayed phase may be more difficult to control than that in the acute phase, and continuous DEX dosing beyond day one may play an important role in preventing delayed nausea. These results support our findings, which suggest that three-day DEX with NK1RA and 5HT3RA may be preferable for patients receiving carboplatin-based chemotherapy, even if they are receiving triplet antiemetic prophylaxis.

We were unable to perform subgroup analysis of patients who received non-carboplatin MEC among the 3-DEX + NK1RA and 1-DEX + NK1RA groups due to insufficient data; thus, the superiority of 3-DEX + NK1RA over 1-DEX + NK1RA in non-carboplatin MEC remains unclear. However, given the benefit of 3-DEX + NK1RA over 1-DEX + NK1RA among the total population, continuous administration of DEX beyond day one may increase the benefit of prophylaxis for delayed-onset CINV in patients receiving non-carboplatin MEC. In particular, because oxaliplatin is known to induce a high incidence of delayed-onset nausea,^45,46^ DEX-sparing regimens should be used with caution in patients receiving oxaliplatin-based chemotherapy.

Delayed-onset nausea is an important part of CINV, and difficult to control.^47-49^ In this NMA, absolute risk difference between 3-DEX + NK1RA and 1-DEX + NK1RA in DP-NN was ≥20%. This result suggests extended DEX dose may play an important role to control delayed-onset nausea in triplet antiemetic prophylaxis with NK1RA. In previous phase III study, NK1RA did not significantly reduce visual analog scale for nausea compared to placebo.^50^ Thus the addition of olanzapine should be considered when limiting administration of DEX to day one in patients with identifiable risk factors for CINV because olanzapine is possibly more effective than NK1RA for preventing nausea.^51-53^

In this NMA, we classified patients who received a three-day dose of a first-generation 5HT3RA or single dose of palonosetron into the long 5HT3RA subgroup to examine them separately from patients who received single-day administration of 5HT3RA, and to set similar conditions for the effect of 5HT3RA on CINV in the delayed phase. In the long 5HT3RA group, 3-DEX + NK1RA showed a nonsignificant superior effect to 1-DEX + NK1RA, and the absolute risk difference between 3-DEX and 1-DEX in CR-DP was 11.4%. The results of a prior RCT suggested that a one-day dose of DEX in combination with palonosetron and NK1RA was an insufficient antiemetic prophylaxis for a cisplatin-containing regimen.^11^ In contrast, a recent study reported that the DEX-sparing strategy in combination with netupitant and palonosetron showed comparable antiemetic effects to four-day DEX in patients receiving cisplatin-based chemotherapy.^54^ Future studies should examine the usefulness of the DEX-sparing strategy in combination with netupitant and palonosetron for carboplatin and non-carboplatin MEC.

Cost is an important factor when selecting antiemetic measures. DEX is less expensive than other antiemetic agents such as palonosetron and NK1RAs. Given that our results suggest there may be considerable benefits to continuous DEX administration, we propose that the majority of patients, with the exception of patients who should receive minimal DEX, such as those intolerant to corticosteroids, should receive DEX beyond day one of treatment.

This NMA has several limitations. First, many of our arguments are based on indirect comparisons between 3-DEX + NK1RA and 1-DEX + NK1RA, which cannot replace the direct comparisons obtained from randomized studies. Second, we could not examine the outcome for those using non-carboplatin MEC due to insufficient data. Further randomized studies are needed to determine the benefits of the DEX-sparing regimen in triplet antiemetic prophylaxis, especially those containing carboplatin and oxaliplatin, which have been shown to benefit from the addition of NK1RA and to lead to a high incidence of delayed-onset CINV.^16,45,46,55^ Third, individual studies in this population are relatively small, which significantly limits statistical power and sensitivity to detect differences. Despite these limitations, our findings, which were derived from available RCT data, highlight concerns related to using the DEX-sparing strategy in combination with NK1RA for the prevention of CINV in patients receiving carboplatin and non-carboplatin MEC.

## Conclusion

Our NMA showed that a three-day dose of DEX with NK1RA tended to have greater antiemetic benefit than a one-day dose of DEX with NK1RA; the absolute risk difference between a three-day and one-day dose of DEX with NK1RA was 9.0% and 24.7% in CR-DP and NN-DP, respectively. Among patients who received carboplatin-based chemotherapy, the absolute risk difference in CR-DP between a three-day and one-day dose of DEX with NK1RA was 12.3%. Therefore, care is needed when choosing the DEX-sparing strategy with NK1RA for patients receiving carboplatin and non-carboplatin MEC. The strategy may be more suitable for selected patients, such as those with few identifiable risk factors for CINV.

## Supplementary Material

oyac060_suppl_Supplementary_Table_S1

oyac060_suppl_Supplementary_Table_S2

oyac060_suppl_Supplementary_Table_S3

oyac060_suppl_Supplementary_Figure_S1

## Data Availability

The data underlying this article are available in the article and in its online supplementary material.

## References

[1] Rowbottom L , Stinson J, McDonald R, et al. Retrospective review of the incidence of monitoring blood glucose levels in patients receiving corticosteroids with systemic anticancer therapy. Ann Palliat Med. 2015;4(2):70-77. https://doi.org/10.3978/j.issn.2224-5820.2015.04.07 25971294 10.3978/j.issn.2224-5820.2015.04.07

[2] Nakamura M , Ishiguro A, Muranaka T, et al. A prospective observational study on effect of short-term periodic steroid premedication on bone metabolism in gastrointestinal cancer (ESPRESSO-01). Oncologist. 2017;22(5):592-600. https://doi.org/10.1634/theoncologist.2016-0308 28341762 10.1634/theoncologist.2016-0308 PMC5423502

[3] Vardy J , Chiew KS, Galica J, et al. Side effects associated with the use of dexamethasone for prophylaxis of delayed emesis after moderately emetogenic chemotherapy. Br J Cancer. 2006;94(7):1011-1015. https://doi.org/10.1038/sj.bjc.6603048 16552437 10.1038/sj.bjc.6603048 PMC2361221

[4] Aapro M , Fabi A, Nolè F, et al. Double-blind, randomised, controlled study of the efficacy and tolerability of palonosetron plus dexamethasone for 1 day with or without dexamethasone on days 2 and 3 in the prevention of nausea and vomiting induced by moderately emetogenic chemotherapy. Ann Oncol. 2010;21(5):1083-1088. https://doi.org/10.1093/annonc/mdp584 20080830 10.1093/annonc/mdp584

[5] Komatsu Y , Okita K, Yuki S, et al. Open-label, randomized, comparative, phase III study on effects of reducing steroid use in combination with Palonosetron. Cancer Sci. 2015;106(7):891-895. https://doi.org/10.1111/cas.12675 25872578 10.1111/cas.12675 PMC4520641

[6] Celio L , Frustaci S, Denaro A, et al. Palonosetron in combination with 1-day versus 3-day dexamethasone for prevention of nausea and vomiting following moderately emetogenic chemotherapy: a randomized, multicenter, phase III trial. Support Care Cancer. 2011;19(8):1217-1225. https://doi.org/10.1007/s00520-010-0941-7 20574663 10.1007/s00520-010-0941-7 PMC3128271

[7] Furukawa N , Kanayama S, Tanase Y, et al. Palonosetron in combination with 1-day versus 3-day dexamethasone to prevent nausea and vomiting in patients receiving paclitaxel and carboplatin. Support Care Cancer. 2015;23(11):3317-3322. https://doi.org/10.1007/s00520-015-2760-3 25947257 10.1007/s00520-015-2760-3

[8] Kosaka Y , Tanino H, Sengoku N, et al. Phase II randomized, controlled trial of 1 day versus 3 days of dexamethasone combined with palonosetron and aprepitant to prevent nausea and vomiting in Japanese breast cancer patients receiving anthracycline-based chemotherapy. Support Care Cancer. 2016;24(3):1405-1411. https://doi.org/10.1007/s00520-015-2905-4 26349772 10.1007/s00520-015-2905-4 PMC4729792

[9] Okada Y , Oba K, Furukawa N, et al. One-day versus three-day dexamethasone in combination with palonosetron for the prevention of chemotherapy-induced nausea and vomiting: a systematic review and individual patient data-based meta-analysis. Oncologist. 2019;24(12):1593-1600. https://doi.org/10.1634/theoncologist.2019-0133 31217343 10.1634/theoncologist.2019-0133 PMC6975929

[10] Celio L , Bonizzoni E, Zattarin E, et al. Impact of dexamethasone-sparing regimens on delayed nausea caused by moderately or highly emetogenic chemotherapy: a meta-analysis of randomised evidence. BMC Cancer. 2019;19(1):1268. https://doi.org/10.1186/s12885-019-6454-y 31888544 10.1186/s12885-019-6454-y PMC6937643

[11] Ito Y , Tsuda T, Minatogawa H, et al. Placebo-controlled, double-blinded phase III study comparing dexamethasone on day 1 with dexamethasone on days 1 to 3 with combined neurokinin-1 receptor antagonist and palonosetron in high-emetogenic chemotherapy. J Clin Oncol. 2018;36(10):1000-1006 https://doi.org/10.1200/JCO.2017.74.4375 29443652 10.1200/JCO.2017.74.4375

[12] NCCN. *NCCN Clinical Practice Guidelines in Oncology Antiemesis Version 1.2021*. Accessed December 23, 2020. https://www.nccn.org/professionals/physician_gls/pdf/antiemesis.pdf.

[13] Aogi K , Takeuchi H, Saeki T, et al. Optimizing antiemetic treatment for chemotherapy-induced nausea and vomiting in Japan: update summary of the 2015 Japan Society of Clinical Oncology Clinical Practice Guidelines for Antiemesis. Int J Clin Oncol. 2021;26(1):1-17. https://doi.org/10.1007/s10147-020-01818-3 33161452 10.1007/s10147-020-01818-3 PMC7788035

[14] Hesketh PJ , Kris MG, Basch E, et al. Antiemetics: ASCO guideline update. J Clin Oncol. 2020;38(24):2782-2797. 32658626 10.1200/JCO.20.01296

[15] Roila F , Warr D, Hesketh PJ, et al. 2016 updated MASCC/ESMO consensus recommendations: prevention of nausea and vomiting following moderately emetogenic chemotherapy. Support Care Cancer. 2017;25(1):289-294. 27510316 10.1007/s00520-016-3365-1

[16] Iihara H , Shimokawa M, Hayashi T, et al. A nationwide, multicenter registry study of antiemesis for carboplatin-based chemotherapy-induced nausea and vomiting in Japan. Oncologist. 2020;25(2):e373-e380. https://doi.org/10.1634/theoncologist.2019-0292 32043774 10.1634/theoncologist.2019-0292 PMC7011617

[17] Hayashi T , Shimokawa M, Mizuki F, et al. Efficacy of one-day versus multiple-day dexamethasone for chemotherapy-induced nausea and vomiting in lung cancer patients receiving carboplatin-based chemotherapy: a propensity score–matched analysis. Support Care Cancer. 2021;29(9):5029-5035. https://doi.org/10.1007/s00520-021-06061-8 33590260 10.1007/s00520-021-06061-8

[18] Zhang J , Carlin BP, Neaton JD, et al. Network meta-analysis of randomized clinical trials: reporting the proper summaries. Clin Trials. 2014;11(2):246-262. 24096635 10.1177/1740774513498322 PMC3972291

[19] Lin L , Zhang J, Hodges JS, et al. Performing arm-based network meta-analysis in R with the pcnetmeta package. J Stat Softw. 2017;80:5. 28883783 10.18637/jss.v080.i05 PMC5584882

[20] Badar T , Cortes J, Borthakur G, et al. Phase II, open label, randomized comparative trial of ondansetron alone versus the combination of ondansetron and aprepitant for the prevention of nausea and vomiting in patients with hematologic malignancies receiving regimens containing high-dose cytara. Biomed Res Int. 2015;2015:1-6. Article ID: 497597. https://doi.org/10.1155/2015/497597 10.1155/2015/497597 PMC4310492 25654108

[21] Di Renzo N , Melillo L, Porretto F, et al. Every-other-day palonosetron plus aprepitant for prevention of emesis following induction chemotherapy for acute myeloid leukemia: a randomized, controlled study from the “Rete Ematologica Pugliese”. Cancer Med. 2020;9(1):170-178. https://doi.org/10.1002/cam4.2628. 31725196 10.1002/cam4.2628 PMC6943081

[22] Hesketh PJ , Wright O, Rosati G, et al. Single-dose intravenous casopitant in combination with ondansetron and dexamethasone for the prevention of oxaliplatin-induced nausea and vomiting: a multicenter, randomized, double-blind, active-controlled, two arm, parallel group study. Support Care Cancer. 2012;20(7):1471-1478. https://doi.org/10.1007/s00520-011-1235-4 21822913 10.1007/s00520-011-1235-4

[23] Kitayama H , Tsuji Y, Sugiyama J, et al. Efficacy of palonosetron and 1-day dexamethasone in moderately emetogenic chemotherapy compared with fosaprepitant, granisetron, and dexamethasone: a prospective randomized crossover study. Int J Clin Oncol. 2015;20(6):1051-1056. https://doi.org/10.1007/s10147-015-0823-6 25822106 10.1007/s10147-015-0823-6

[24] Schmitt T , Goldschmidt H, Neben K, et al. Aprepitant, granisetron, and dexamethasone for prevention of chemotherapy-induced nausea and vomiting after high-dose melphalan in autologous transplantation for multiple myeloma: results of a randomized, placebo-controlled phase III trial. J Clin Oncol. 2014;32(30):3413-3420. https://doi.org/10.1200/jco.2013.55.0095 25225424 10.1200/JCO.2013.55.0095

[25] Xiong J , Zhao G, Yang S, et al. Efficacy, tolerability and pharmacokinetic impact of aprepitant in sarcoma patients receiving ifosfamide and doxorubicin chemotherapy: a randomized controlled trial. Adv Ther. 2019;36(2):355-364. https://doi.org/10.1007/s12325-018-0862-2 30607545 10.1007/s12325-018-0862-2

[26] Inoue A , Yamada Y, Matsumura Y, et al. Randomized study of dexamethasone treatment for delayed emesis, anorexia and fatigue induced by irinotecan. Support Care Cancer. 2003;11(8):528-532. https://doi.org/10.1007/s00520-003-0488-y 12844250 10.1007/s00520-003-0488-y

[27] Aridome K , Mori S-I, Baba K, et al. A phase II, randomized study of aprepitant in the prevention of chemotherapy-induced nausea and vomiting associated with moderately emetogenic chemotherapies in colorectal cancer patients. Mol Clin Oncol. 2016;4(3):393-398. https://doi.org/10.3892/mco.2015.724 26998290 10.3892/mco.2015.724 PMC4774568

[28] Celio L , Frustaci S, Denaro A, et al. Palonosetron in combination with 1-day versus 3-day dexamethasone for prevention of nausea and vomiting following moderately emetogenic chemotherapy: a randomized, multicenter, phase III trial. Support Care Cancer. 2011;19(8):1217-1225 20574663 10.1007/s00520-010-0941-7 PMC3128271

[29] Furukawa N , Kanayama S, Tanase Y, et al. Palonosetron in combination with 1-day versus 3-day dexamethasone to prevent nausea and vomiting in patients receiving paclitaxel and carboplatin. Support Care Cancer. 2015;23(11):3317-3322. https://doi.org/10.1007/s00520-015-2760-3 25947257 10.1007/s00520-015-2760-3

[30] Ito Y , Karayama M, Inui N, et al. Aprepitant in patients with advanced non-small-cell lung cancer receiving carboplatin-based chemotherapy. Lung Cancer. 2014;84(3):259-264. https://doi.org/10.1016/j.lungcan.2014.03.017 24746177 10.1016/j.lungcan.2014.03.017

[31] Kaushal P , Atri R, Soni A, et al. Comparative evaluation of triplet antiemetic schedule versus doublet antiemetic schedule in chemotherapy-induced emesis in head and neck cancer patients. Ecancermedicalscience. 2015;9:567. https://doi.org/10.3332/ecancer.2015.567 26435740 10.3332/ecancer.2015.567 PMC4583242

[32] Kim JE , Jang JS, Kim JW, et al. Efficacy and safety of aprepitant for the prevention of chemotherapy-induced nausea and vomiting during the first cycle of moderately emetogenic chemotherapy in Korean patients with a broad range of tumor types. Support Care Cancer. 2017;25(3):801-809. https://doi.org/10.1007/s00520-016-3463-0 27826874 10.1007/s00520-016-3463-0

[33] Kusagaya H , Inui N, Karayama M, et al. Evaluation of palonosetron and dexamethasone with or without aprepitant to prevent carboplatin-induced nausea and vomiting in patients with advanced non-small-cell lung cancer. Lung Cancer. 2015;90(3):410-416. https://doi.org/10.1016/j.lungcan.2015.11.009 26791800 10.1016/j.lungcan.2015.11.009

[34] Maehara M , Ueda T, Miyahara D, et al. Clinical efficacy of aprepitant in patients with gynecological cancer after chemotherapy using paclitaxel and carboplatin. Anticancer Res. 2015;35(8): 4527-4534. 26168497

[35] Matsuura M , Satohisa S, Teramoto M, et al. Palonosetron in combination with 1-day versus 3-day dexamethasone for prevention of nausea and vomiting following paclitaxel and carboplatin in patients with gynecologic cancers: a randomized, multicenter, phase-II trial. J Obstet Gynaecol Res. 2015;41(10):1607-1613. https://doi.org/10.1111/jog.12748 26199182 10.1111/jog.12748

[36] Nishimura J , Satoh T, Fukunaga M, et al. Combination antiemetic therapy with aprepitant/fosaprepitant in patients with colorectal cancer receiving oxaliplatin-based chemotherapy (SENRI trial): a multicentre, randomised, controlled phase 3 trial. Eur J Cancer. 2015;51(10):1274-1282. https://doi.org/10.1016/j.ejca.2015.03.024 25922233 10.1016/j.ejca.2015.03.024

[37] Rapoport BL , Jordan K, Boice JA, et al. Aprepitant for the prevention of chemotherapy-induced nausea and vomiting associated with a broad range of moderately emetogenic chemotherapies and tumor types: a randomized, double-blind study. Support Care Cancer. 2010;18(4):423-431. https://doi.org/10.1007/s00520-009-0680-9 19568773 10.1007/s00520-009-0680-9

[38] Schwartzberg LS , Modiano MR, Rapoport BL, et al. Safety and efficacy of rolapitant for prevention of chemotherapy-induced nausea and vomiting after administration of moderately emetogenic chemotherapy or anthracycline and cyclophosphamide regimens in patients with cancer: a randomised, active-controlled. Lancet Oncol. 2015;16(9):1071-1078. https://doi.org/10.1016/S1470-2045(15)00034-0 26272768 10.1016/S1470-2045(15)00034-0

[39] Sugimori Y , Ota T, Ujihira T, et al. A phase II randomised study to evaluate the efficacy of aprepitant plus palonosetron for preventing delayed-phase CINV associated with TC therapy in gynaecological cancer. J Obstet Gynaecol Res. 2017;43(9):1454-1459. https://doi.org/10.1111/jog.13378 28952201 10.1111/jog.13378

[40] Tanioka M , Kitao A, Matsumoto K, et al. A randomised, placebo-controlled, double-blind study of aprepitant in nondrinking women younger than 70 years receiving moderately emetogenic chemotherapy. Br J Cancer. 2013;109(4):859-865. https://doi.org/10.1038/bjc.2013.400 23860530 10.1038/bjc.2013.400 PMC3749572

[41] Weinstein C , Jordan K, Green SA, et al. Single-dose fosaprepitant for the prevention of chemotherapy-induced nausea and vomiting associated with moderately emetogenic chemotherapy: results of a randomized, double-blind phase III trial. Ann Oncol. 2016;27(1):172-178. https://doi.org/10.1093/annonc/mdv482 26449391 10.1093/annonc/mdv482 PMC4684151

[42] Yahata H , Kobayashi H, Sonoda K, et al. Efficacy of aprepitant for the prevention of chemotherapy-induced nausea and vomiting with a moderately emetogenic chemotherapy regimen: a multicenter, placebo-controlled, double-blind, randomized study in patients with gynecologic cancer receiving paclitaxel and carboplatin. Int J Clin Oncol. 2016;21(3):491-497. https://doi.org/10.1007/s10147-015-0928-y 26662632 10.1007/s10147-015-0928-y

[43] Hesketh PJ , Schnadig ID, Schwartzberg LS, et al. Efficacy of the neurokinin-1 receptor antagonist rolapitant in preventing nausea and vomiting in patients receiving carboplatin-based chemotherapy. Cancer. 2016;122(15):2418-2425. https://doi.org/10.1002/cncr.30054 27176138 10.1002/cncr.30054 PMC5084806

[44] Roila F , Herrstedt J, Aapro M, et al. Guideline update for MASCC and ESMO in the prevention of chemotherapy- and radiotherapy-induced nausea and vomiting: results of the Perugia consensus conference. Ann Oncol. 2010;21:v232-v243. https://doi.org/10.1093/annonc/mdq194 20555089 10.1093/annonc/mdq194

[45] Tamura K , Aiba K, Saeki T, et al. Testing the effectiveness of antiemetic guidelines: results of a prospective registry by the CINV Study Group of Japan. Int J Clin Oncol. 2015;20(5):855-865. https://doi.org/10.1007/s10147-015-0786-7 25681876 10.1007/s10147-015-0786-7

[46] Tsuji Y , Baba H, Takeda K, et al. Chemotherapy-induced nausea and vomiting (CINV) in 190 colorectal cancer patients: a prospective registration study by the CINV study group of Japan. Expert Opin Pharmacother. 2017;18(8):753-758. https://doi.org/10.1080/14656566.2017.1317746 28395603 10.1080/14656566.2017.1317746

[47] Ng T , Mazzarello S, Wang Z, et al. Choice of study endpoint significantly impacts the results of breast cancer trials evaluating chemotherapy-induced nausea and vomiting. Breast Cancer Res Treat. 2016;155(2):337-344. https://doi.org/10.1007/s10549-015-3669-8 26732944 10.1007/s10549-015-3669-8

[48] Ng TL , Hutton B, Clemons M. Chemotherapy-induced nausea and vomiting: time for more emphasis on nausea? Oncologist. 2015;20(6):576-583. https://doi.org/10.1634/theoncologist.2014-0438 25948677 10.1634/theoncologist.2014-0438 PMC4571780

[49] Hernandez Torres C , Mazzarello S, Ng T, et al. Defining optimal control of chemotherapy-induced nausea and vomiting-based on patients’ experience. Support Care Cancer. 2015;23(11):3341-3359. https://doi.org/10.1007/s00520-015-2801-y 26108169 10.1007/s00520-015-2801-y

[50] Albany C , Brames MJ, Fausel C, et al. Randomized, double-blind, placebo-controlled, phase III cross-over study evaluating the oral neurokinin-1 antagonist aprepitant in combination with a 5HT3 receptor antagonist and dexamethasone in patients with germ cell tumors receiving 5-day cisplatin combination chemotherapy regimens: a Hoosier Oncology Group study. J Clin Oncol. 2012;30(32):3998-4003. https://doi.org/10.1200/JCO.2011.39.5558 22915652 10.1200/JCO.2011.39.5558

[51] Tan L , Liu J, Liu X, et al. Clinical research of Olanzapine for prevention of chemotherapy-induced nausea and vomiting. J Exp Clin Cancer Res. 2009;28(1):131. https://doi.org/10.1186/1756-9966-28-131 19775450 10.1186/1756-9966-28-131 PMC2761865

[52] Chow R , Chiu L, Navari R, et al. Efficacy and safety of olanzapine for the prophylaxis of chemotherapy-induced nausea and vomiting (CINV) as reported in phase I and II studies: a systematic review. Support Care Cancer. 2016;24(2):1001-1008. https://doi.org/10.1007/s00520-015-3000-6 26530228 10.1007/s00520-015-3000-6

[53] Navari RM , Gray SE, Kerr AC, et al. Olanzapine versus aprepitant for the prevention of chemotherapy-induced nausea and vomiting: a randomized phase III trial. J Support Oncol. 2011;9(5):188-195. https://doi.org/10.1016/j.suponc.2011.05.002 22024310 10.1016/j.suponc.2011.05.002

[54] Celio L , Cortinovis D, Cogoni AA, et al. Dexamethasone-sparing regimens with oral netupitant and palonosetron for the prevention of emesis caused by high-dose cisplatin: a randomized noninferiority study. Oncologist. 2021;26(10):e1854-e1861. https://doi.org/10.1002/onco.13851 34101934 10.1002/onco.13851 PMC8488764

[55] Hayashi T , Shimokawa M, Matsuo K, et al. 5HT3RA plus dexamethasone plus aprepitant for controlling delayed chemotherapy-induced nausea and vomiting in colorectal cancer. Cancer Sci. 2021;112(2):744-750. https://doi.org/10.1111/cas.14757 33274555 10.1111/cas.14757 PMC7893986

