# Supplementary material for: One-Day Versus Three-Day Dexamethasone with NK1RA for Patients Receiving Carboplatin and Moderate Emetogenic Chemotherapy: A Network Meta-analysis
Source: Oncologist. 2022 Apr 15;27(6):e524–32. doi: 10.1093/oncolo/oyac060 (PMC9177112; doi:10.1093/oncolo/oyac060)
Supplement: oyac060_suppl_Supplementary_Table_S1 [file oyac060_suppl_supplementary_table_s1.docx]

Supplementary Table 1. Search terms

| #1 | (“nausea”[TW] OR “vomiting”[TW] OR “nausea or vomiting”[TW] OR “chemotherapy-induced nausea or vomiting”[TW] OR “complete control”[TW] OR “total control”[TW] OR “complete response”[TW]) AND (“dexamethasone”[TIAB] OR “dexamethasone sparing”[TW] OR “dexamethasone-sparing”[TW] OR (“dexamethasone”[TW] AND “one-day”[TW]) OR (“dexamethasone”[TW] AND “single day”[TW])) AND (“moderate emetic risk”[TW] OR “MEC”[TW] OR “aldesleukin”[TW] OR “amifostine”[TW] OR “azacitidine”[TW] OR “bendamustine”[TW] OR “busulfan”[TW] OR “carboplatin”[TW] OR “carmustine”[TW] OR “clofarabine”[TW] OR “cyclophosphamide”[TW] OR “cytarabine”[TW] OR “dactinomycin”[TW] OR “daunorubicin”[TW] OR “dinutuximab”[TW] OR “doxorubicin”[TW] OR “epirubicin”[TW] OR “trastuzumab deruxtecan”[TW] OR “idarubicine”[TW] OR “ifosfamide”[TW] OR “irinotecan”[TW] OR “lurbinectedin”[TW] OR “melphalan”[TW] OR “methotrexate”[TW] OR “oxaliplatin”[TW] OR “temozolomide”[TW] OR “trabectedin”[TW]) NOT (“olanzapine”[TI] OR “multiple myeloma”[mh] OR “lymphoma”[mh] OR “rituximab”[TIAB] OR “amyloidosis”[mh] OR “leukemia”[mh]) |
| --- | --- |
| #2 | (“nausea”[TW] OR “vomiting”[TW] OR “nausea or vomiting”[TW] OR “chemotherapy-induced nausea or vomiting”[TW] OR “complete control”[TW] OR “total control”[TW] OR “complete response”[TW]) AND (“MK-0869”[TW] OR “L-754,030”[TW] OR “CJ-11,974”[TW] OR “fosaprepitant”[TW] OR “casopitant”[TW] OR “ezlopitant”[TW] “NK-1”[TW] OR “NK1”[TW] OR “neurokinin-1”[TW] OR “netupitant”[TW] OR “rolapitant”[TW] OR “aprepitant”[TW]) AND (“moderate emetic risk”[TW] OR “MEC”[TW] OR “aldesleukin”[TW] OR “amifostine”[TW] OR “azacitidine”[TW] OR “bendamustine”[TW] OR “busulfan”[TW] OR “carboplatin”[TW] OR “carmustine”[TW] OR “clofarabine”[TW] OR “cyclophosphamide”[TW] OR “cytarabine”[TW] OR “dactinomycin”[TW] OR “daunorubicin”[TW] OR “dinutuximab”[TW] OR “doxorubicin”[TW] OR “epirubicin”[TW] OR “trastuzumab deruxtecan”[TW] OR “idarubicine”[TW] OR “ifosfamide”[TW] OR “irinotecan”[TW] OR “lurbinectedin”[TW] OR “melphalan”[TW] OR “methotrexate”[TW] OR “oxaliplatin”[TW] OR “temozolomide”[TW] OR “trabectedin”[TW]) NOT “olanzapine”[TI] |
| #3 | #1 OR #2 |
