# Supplementary material for: One-Day Versus Three-Day Dexamethasone with NK1RA for Patients Receiving Carboplatin and Moderate Emetogenic Chemotherapy: A Network Meta-analysis
Source: Oncologist. 2022 Apr 15;27(6):e524–32. doi: 10.1093/oncolo/oyac060 (PMC9177112; doi:10.1093/oncolo/oyac060)
Supplement: oyac060_suppl_Supplementary_Table_S2 [file oyac060_suppl_supplementary_table_s2.docx]

|  | Randomization process | Deviations from intended interventions | Missing outcome data | Measurement of the outcome | Selection of the reported result | Overall bias |
| --- | --- | --- | --- | --- | --- | --- |
| Aridome_2016 | Low | Low | Low | Low | Low | Low |
| Celio_2011 | Low | Low | Low | Low | Low | Low |
| Furukawa_2015 | Low | Low | Low | Low | Low | Low |
| Ito_2014 | Low | Low | Low | Low | Low | Low |
| Kaushal_2015 | Low | Low | Low | Low | Low | Low |
| Kim_2017 | Low | Low | Low | Low | Low | Low |
| Komatsu_2015 | Low | Low | Low | Low | Low | Low |
| Maehara_2015 | Low | Low | Low | Low | Low | Low |
| Matsuura_2015 | Low | Low | Low | Low | Low | Low |
| Nishimura_2015 | Low | Low | Low | Low | Low | Low |
| Rapoport_2010 | Low | Low | Low | Low | Low | Low |
| Schwartzberg_2015 | Low | Low | Low | Low | Low | Low |
| Sugimori_2017 | Some concerns | Low | Low | Low | Low | Some concerns |
| Tanioka_2013 | Low | Low | Low | Low | Low | Low |
| Weinstein_2016 | Low | Low | Low | Low | Low | Low |
| Yahata_2016 | Low | Low | Low | Low | Low | Low |

Supplementary Table 2. Risk of bias
