# Supplementary material for: One-Day Versus Three-Day Dexamethasone with NK1RA for Patients Receiving Carboplatin and Moderate Emetogenic Chemotherapy: A Network Meta-analysis
Source: Oncologist. 2022 Apr 15;27(6):e524–32. doi: 10.1093/oncolo/oyac060 (PMC9177112; doi:10.1093/oncolo/oyac060)
Supplement: oyac060_suppl_Supplementary_Table_S3 [file oyac060_suppl_supplementary_table_s3.docx]

Supplemental Table 3. Number of included studies and heterogeneity I2 statistics for each outcome

Complete response during the delayed phase among the entire population

| Antiemetic regimen | No. of studies (no. of participants) | Heterogeneity I2 statistic |
| --- | --- | --- |
| 3-DEX+NK1RA | 8 (478) | 57.3% |
| 1-DEX+NK1RA | 5 (1438) | 72.7% |
| 3-DEX | 12 (816) | 53.0% |
| 1-DEX | 9 (1758) | 68.5% |

No nausea during the delayed phase among the entire population

| Antiemetic regimen | No. of studies (no. of participants) | Heterogeneity I2 statistic |
| --- | --- | --- |
| 3-DEX+NK1RA | 6 (392) | 43.5% |
| 1-DEX+NK1RA | 1 (151) | NA |
| 3-DEX | 6 (385) | 74.6% |
| 1-DEX | 1 (146) | NA |

Complete response during the delayed phase among those who received a carboplatin-based regimen

| Antiemetic regimen | No. of studies (no. of participants) | Heterogeneity I2 statistic |
| --- | --- | --- |
| 3-DEX+NK1RA | 5 (187) | 8.1% |
| 1-DEX+NK1RA | 3 (600) | 88.2% |
| 3-DEX | 7 (279) | 28.3% |
| 1-DEX | 5 (710) | 67.6% |

Complete response during the delayed phase among those treated with a three-day dose of first-generation 5HT3RA or single dose of palonosetron

| Antiemetic regimen | No. of studies (no. of participants) | Heterogeneity I2 statistic |
| --- | --- | --- |
| 3-DEX+NK1RA | 3 (110) | 52.4% |
| 1-DEX+NK1RA | 1 (322) | NA |
| 3-DEX | 7 (454) | 49.3% |
| 1-DEX | 8 (1612) | NA |

Abbreviations

1-DEX: one-day dexamethasone; 1-DEX+NK1RA: one-day dexamethasone with neurokinin-1 receptor antagonist; 3-DEX: three-day dexamethasone; 3-DEX+NK1RA: three-day dexamethasone with neurokinin-1 receptor antagonist; 5-HT3RA: 5-hydroxytryptamine-3 receptor antagonist; NA: not assessed
