# Supplementary figures and images for: One-Day Versus Three-Day Dexamethasone with NK1RA for Patients Receiving Carboplatin and Moderate Emetogenic Chemotherapy: A Network Meta-analysis
Source: Oncologist. 2022 Apr 15;27(6):e524–32. doi: 10.1093/oncolo/oyac060 (PMC9177112; doi:10.1093/oncolo/oyac060)

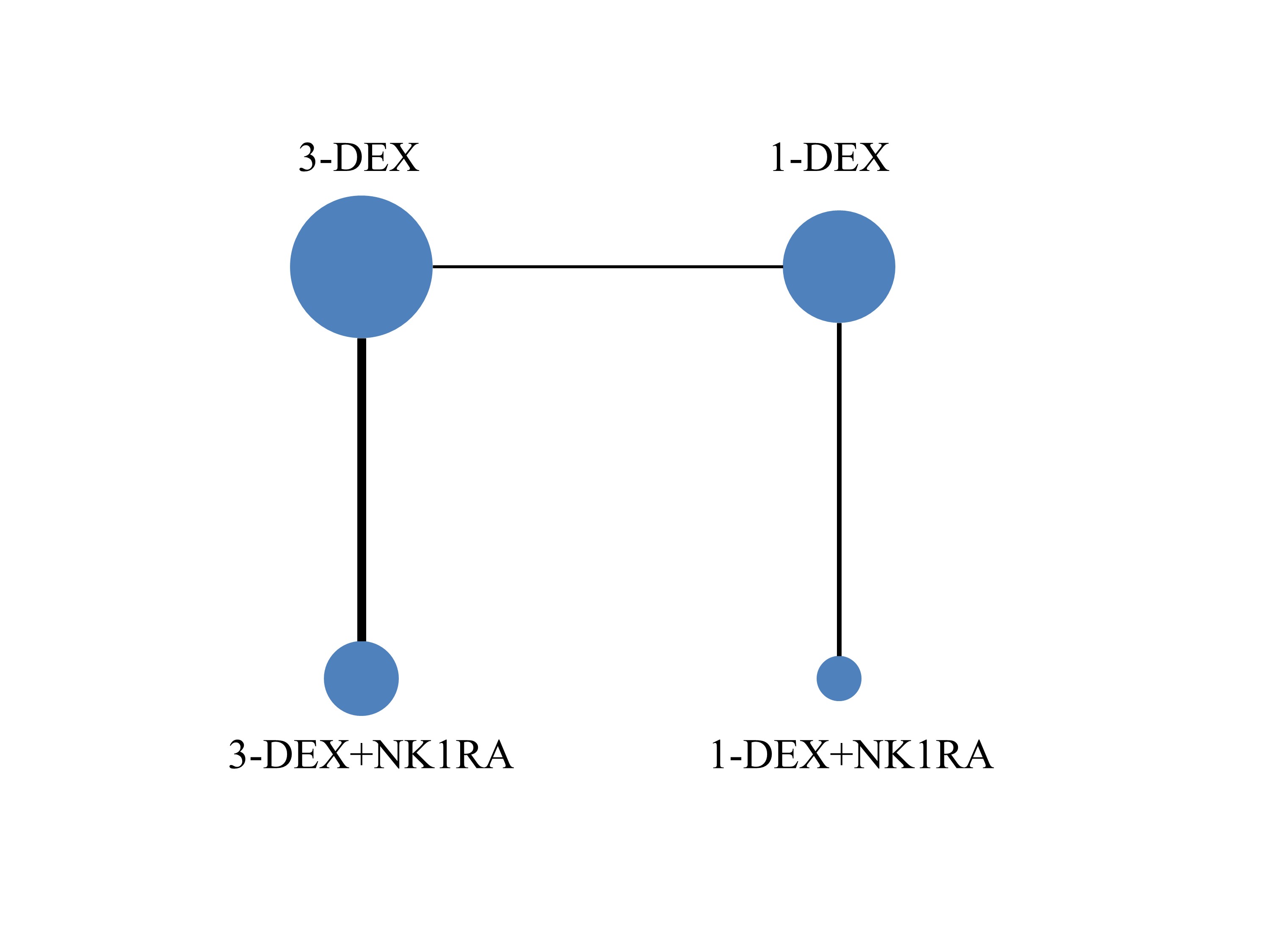

Supplement: oyac060_suppl_Supplementary_Figure_S1 [file oyac060_suppl_supplementary_figure_s1.jpeg]
